# Extended Postnatal Brain Development in the Longest-Lived Rodent: Prolonged Maintenance of Neotenous Traits in the Naked Mole-Rat Brain

**DOI:** 10.3389/fnins.2016.00504

**Published:** 2016-11-08

**Authors:** Miranda E. Orr, Valentina R. Garbarino, Angelica Salinas, Rochelle Buffenstein

**Affiliations:** ^1^Department of Physiology, University of Texas Health Science Center at San AntonioSan Antonio, TX, USA; ^2^The Barshop Institute for Longevity, Aging Studies, University of Texas Health Science Center at San AntonioSan Antonio, TX, USA; ^3^Calico Life Sciences LLCSouth San Francisco, CA, USA

**Keywords:** naked mole-rat, *Heterocephalus glaber*, neurogenesis, synaptogenesis, comparative biology, tau, neoteny

## Abstract

The naked mole-rat (NMR) is the longest-lived rodent with a maximum lifespan >31 years. Intriguingly, fully-grown naked mole-rats (NMRs) exhibit many traits typical of neonatal rodents. However, little is known about NMR growth and maturation, and we question whether sustained neotenous features when compared to mice, reflect an extended developmental period, commensurate with their exceptionally long life. We tracked development from birth to 3 years of age in the slowest maturing organ, the brain, by measuring mass, neural stem cell proliferation, axonal, and dendritic maturation, synaptogenesis and myelination. NMR brain maturation was compared to data from similar sized rodents, mice, and to that of long-lived mammals, humans, and non-human primates. We found that at birth, NMR brains are significantly more developed than mice, and rather are more similar to those of newborn primates, with clearly laminated hippocampi and myelinated white matter tracts. Despite this more mature brain at birth than mice, postnatal NMR brain maturation occurs at a far slower rate than mice, taking four-times longer than required for mice to fully complete brain development. At 4 months of age, NMR brains reach 90% of adult size with stable neuronal cytostructural protein expression whereas myelin protein expression does not plateau until 9 months of age in NMRs, and synaptic protein expression continues to change throughout the first 3 years of life. Intriguingly, NMR axonal composition is more similar to humans than mice whereby NMRs maintain expression of three-repeat (3R) tau even after brain growth is complete; mice experience an abrupt downregulation of 3R tau by postnatal day 8 which continues to diminish through 6 weeks of age. We have identified key ages in NMR cerebral development and suggest that the long-lived NMR may provide neurobiologists an exceptional model to study brain developmental processes that are compressed in common short-lived laboratory animal models.

## Introduction

The naked mole-rat (NMR; Rodentia: Heterocephalidae, *Heterocephalus glaber*) is the longest-lived rodent known (Buffenstein, 2005; Edrey et al., 2011). Adult NMRs exhibit attenuated age-related changes in reproductive and cardiac function, and maintain body composition and metabolism well into their third decade of life revealing a negligible senescence phenotype (Buffenstein, 2008; Edrey et al., 2011). Curiously, full-grown NMRs display many traits that are considered neotenous when compared to other rodents (Maina et al., 1992; Buffenstein, 2001). Some of these traits may be ecophysiological adaptations to life in underground, sealed, hypoxic, and hypercapnic burrows (Buffenstein, 1996; Kim et al., 2011; Fang et al., 2014; Garbarino et al., 2015), a niche inhabited since the early Miocene. Moreover, many of these traits are shared among the two African mole-rat families (Bathyergidae and Heterocephalidae) as well as the more distant Asian and Middle Eastern mole-rats (Faulkes et al., 2004; Upham and Patterson, 2012). For example, like neonates in general, adult NMRs are extremely tolerant of hypoxia (Larson and Park, 2009). This has been attributed to their retained expression of a neonatal NMDA receptor subunit that is less sensitive to hypoxic conditions (Peterson et al., 2012a,b). Other neotenous traits include unfused growth plates in bone (Pinto et al., 2010), immature lung morphology (Maina et al., 1992). Moreover, many of the described NMR brain features are suggestive of an extended period of development or persistent neoteny as well. These include blunted neuronal calcium response (Peterson et al., 2012a); sustained presence of the neurotrophic growth factor, neuregulin (Edrey et al., 2012); beta-amyloid (Edrey et al., 2013); phosphorylated tau (Orr et al., 2015); and neuroplasticity markers, doublecortin and PSA-NCAM (Penz et al., 2015).

Humans display many neotenous traits when compared to chimpanzees (Gould, 1977; Alberch et al., 1979; Penin et al., 2002; Mitteroecker et al., 2004), but both species mature into adults with traits unique from their developmental period. We propose that the continued expression of neotenous traits in full-grown NMRs when compared to short-lived rodents reflects peramorphosis, or delayed maturation and extended developmental periods. The NMR gestation period extends nearly 3.5 times longer than mice, 71 and 21 days, respectively (Jarvis, 1991) and although NMRs can attain sexual maturity and breed at 6 months of age, NMR somatic growth continues well into their first year of life (O'Riain and Jarvis, 1998), providing evidence that NMRs require more developmental time to mature than mice. Until now, however, an effort to understand NMR brain development has not been undertaken making it difficult to determine appropriate ages for comparative studies. Not surprisingly, therefore, in the few studies focused on NMR neurobiology, neurogenesis, and brain plasticity, “young” NMRs range in age from a couple months to 5 years old (Holmes et al., 2007, 2011; Peragine et al., 2014; Penz et al., 2015). Here our goal is to provide a comprehensive report detailing NMR brain development specifically compared to mice. In doing so, we provide data for investigators to make better-informed decisions when choosing experimental ages and to better understand certain caveats that need to be taken into consideration when using NMRs. Ultimately this will result in a more accurate representation of experimental findings, and provide scientific rationale for some of the previously “unexplained quirks” of NMRs, many of which can be explained by differences in brain development. We have specifically chosen to compare NMRs with mice, rather than with more closely related species that may show greater similarities. Not only are mice and mole-rats similar sized rodents enabling us to highlight species differences not directly linked to allometry, but the mouse has been extremely well characterized, serving firstly as an experimental control with which to evaluate the various techniques used and thereafter enabling the evaluation of developmental features of this potentially biomedically important, albeit poorly characterized, non-traditional model for biomedical research.

Recently we reported that brain tau phosphorylation decreased precipitously during the first 6 months of NMR postnatal life; nevertheless, they still expressed higher levels than mice (Orr et al., 2015). Since tau phosphorylation and expression signify different states of neuronal developmental maturity and stability (Goedert et al., 1989a,b; Kosik et al., 1989; Goedert and Jakes, 1990; Ksiezak-Reding et al., 1992; Bramblett et al., 1993; Brion et al., 1993; Kenessey and Yen, 1993; Köpke et al., 1993), we hypothesize that 6 months of age may mark a major milestone in NMR brain development. To test this, we examined brain maturation throughout the first 3 years of NMR postnatal life using brain and body mass measures and established markers of neural stem cell proliferation, axon, and dendritic maturation, synaptogenesis, and myelination. These enabled us to define key stages in NMR brain maturation. We found that NMRs do not follow the rapid brain maturation trajectory analogous to similar-sized rodents, but instead more closely track the extended brain development observed in other long-lived species, including humans. Here we present the NMR as a highly suited model to study brain developmental processes and reveal that observed differences from their rodent laboratory relatives (mice) seemingly are not unique to NMRs, but instead common traits of long-lived species.

## Materials and methods

### Animals

NMRs have complex behavior associated with eusociality (Jarvis, 1981), exhibiting a division of labor such that breeding is restricted to only one dominant female and three or four males within the colony. Should she die or be removed, the remaining females will fight to death to establish dominance and reactivate the reproduction endocrine axis. This feature, while common in the eusocial insects, is atypical of mammals, with eusociality only evident in the African mole-rat families. This coupled with the extended gestation period (71 days) render collecting brains from each gestational age not feasible. We therefore designed this study to comprehensively examine postnatal, rather than *in utero*, brain development. For species comparison, we reference literature from decades of well-established studies on cerebral brain development in mice, rats, humans, and non-human primates. We only present new data for mice when antibody validation is required (i.e., histological interspecies comparison).

NMRs were housed in multichambered plexiglass burrow systems at 28–30°C and 30–50% relative humidity similar to their native habitat. NMRs were kept on a 12-h light/dark cycle and supplied *ad-libitum* with fruit and vegetables, supplemented with a high protein and vitamin enriched cereal (Pronutro, South Africa). UTHSCSA is fully accredited by International AAALAC (Association for Assessment and Accreditation of Laboratory Animal Care). All studies were carried out in accordance with the ethical standards of the Guide for the Care and Use of Laboratory Animals of the National Institutes of Health, U.S. Public Health Service and approved by the Institutional Animal Care and Use Committee (IACUC); protocol #07123.

### Tissue collection

Newborn and postnatal NMRs and C57BL/6 mice ranging in age from newborn (day 0) through 3 years were used. Neonatal animals were sacrificed by decapitation and adults were anesthetized by isoflurane inhalation prior to decapitation. Brains were immediately harvested, weighed and sagitally bisected. One hemibrain was drop fixed in 10% zinc formalin for 48 h, then transferred to PBS containing 0.02% sodium azide and stored at 4°C until used in histological processing. The other hemibrain was snap-frozen in liquid nitrogen and stored at −80°C for future biochemical analyses.

### Tissue homogenization

For biochemistry, frozen cerebrum (hemibrain lacking cerebellum, olfactory bulb and brain stem) were thawed slightly on ice and mechanically homogenized with dounce and pestle in ice-cold Buffer H (10 mM Tris HCl pH 7.4, 1 mM EGTA, 0.8 M NaCl, 10% sucrose) containing complete protease inhibitor (Roche, Basel, Switzerland) with phosphatase inhibitors (Invitrogen, Carlsbad, CA). Half of the lysate was then sonicated with 10-s pulses (speed 5) (60 Sonic Dismembrator, Fisher Scientific). Both sonicated and dounce homogenized brain homogenates were centrifuged 14,000 rpm for 20 min at 4°C. The supernatant protein concentrations were determined using BCA assay (Pierce, Rockford, IL). Supernatant protein concentration of 0.2 μg/μL was used for capillary electrophoresis.

### Capillary electrophoresis immunoassay

Capillary electrophoresis (CE) immunoblotting, or Simple Western analyses, were performed using Wes™ platform according to the manufacturer's protocol (ProteinSimple Santa Clara, CA) and described previously (Orr et al., 2015). Simple Western analysis is carried out at room temperature, and instrument default settings were used. Table 1 provides a complete list of antibodies used for capillary electrophoresis. Control capillaries containing brain homogenates with antibody diluent as the primary antibody were used to assess non-specific binding of secondary antibody; non-specific binding was not observed with either mouse or rabbit secondary antibodies. For each antibody, antibody dilutions ranging from 1:25 to 1:200 was assessed to determine optimal antibody concentration with positive control samples (mouse brain homogenates). For antibodies with high affinity binding, additional primary antibody dilutions ranging from 1:200 to 1:1000 were tested. Sonicated samples were used to assess levels of synaptophysin (Cell Signaling, Cat# 5461S); PSD95 (Abcam, Cat# ab2723); myelin associated glycoprotein (MAG) (Cell Signaling, Cat#: 9043P); myelin basic protein (MBP) (Cell Signaling, 13344S). Dounce homogenized samples were used for NF-L (Cell Signaling, Cat# 2837P); RD3 (Millipore, Cat#: 05-803); RD4 (Millipore, Cat#: 05-804); tyrosine hydroxylase (TH) (Cell Signaling, Cat#: 2792); 4G11 (rPeptide, Cat#: TA-1001) and Map2 (Cell Signaling, Cat#: 4542). The digital images were analyzed with Compass software (ProteinSimple, Santa Clara, CA), and the quantified data of the detected protein were reported as molecular weight. Protein densitometry was calculated by dividing the area under the curve of each protein of interest by area under the curve of β-actin loading control mouse (Cell Signaling, Cat#: 3700) or rabbit (Cell Signaling, Cat#: 4970). See Table 1 for detailed description of antibody immunogen and dilutions.

**Table 1:** **Antibodies used in this study**.

**Antibody**	**Immunogen**	**Source**	**Dilution**
Anti-Synaptophysin	Synthetic peptide surrounding Ala230 of human synaptophysin.	Cell Signaling Technology, 5461S, rabbit polyclonal	1:50 CE
Anti-PSD95	Purified recombinant rat PSD-95.	Abcam, ab2723, mouse monoclonal	1:200 CE
Anti-MAG	Synthetic peptide surrounding Arg605 of human MAG protein.	Cell Signaling Technology, 9043P, rabbit monoclonal	1:100 CE; 1:800 IHC
Anti-Myelin Basic Protein (MBP)	Recombinant protein specific to human MBP.	Cell Signaling Technology, 13344S, mouse monoclonal	1:100 CE
Anti- Neurofilament-L (NF-L)	Synthetic peptide surrounding Glu450 of human NF-L	Cell Signaling Technology, 2837P, rabbit monoclonal	1:500 CE
Anti-Tau (4-repeat isoforms)	Synthetic N279D 4R-tau peptide (VQIIDKKLDLSNVQSKC)	Cosmo Bio Co. TIP-4RT-P01, rabbit polyclonal	1:50 CE
Anti-Tau (3-repeat isoform RD3, clone 8E6/C11)	Bovine thyroglobulin conjugated synthetic peptide of human tau amino acids 209–224.	Millipore, 05–803, mouse, monoclonal	1:500 CE
Anti-Tyrosine Hydroxylase (TH)	Synthetic peptide of amino-terminal of human TH.	Cell Signaling Technology, 2792, rabbit polyclonal	1:50 CE
Anti-Tau 4G11	57–64 of the full length Tau isoform (Tau-441)	rPeptide, TA-1001, mouse monoclonal	1:100 CE
Anti-MAP2	Synthetic peptide of human MAP2 carboxy-terminal residues.	Cell Signaling Technology, 4542, rabbit polyclonal	1:100 CE
Anti-β-Actin	Synthetic peptide of human β-actin protein amino-terminal residues	Cell Signaling Technology, 4970, rabbit monoclonal	1:100 CE
Anti-β-Actin	Synthetic peptide of human β-actin protein amino-terminal residues	Cell Signaling Technology, 3700, mouse monoclonal	1:200 CE
Anti-Doublecortin (DCX)	Epitope mapping at the C-terminus of human DCX	Santa Cruz Biotechnology, sc-8066, mouse monoclonal	1:1000 IHC
Anti-Sox-2	Epitope mapping near the C-terminus of human Sox-2.	Santa Cruz Biotechnology, sc-17320, mouse monoclonal	1:500 IHC

### Immunohistochemistry

Zinc formalin fixed tissues were sectioned (30 μm thick) using a sliding vibratome, and stored in 0.02% sodium azide in PBS until immunostaining was conducted. The endogenous peroxidase activity was quenched with 3% H_2_O_2_ in 10% methanol for 30 min. Sections were washed in Tris buffered saline (pH 7.4) and incubated in biotinylated secondary antibody for 1 h at 20°C. Sections were developed with diaminobenzidine substrate using the avidin-biotin horseradish peroxidase system (Vector Labs, Burlingame, CA, USA). An additional step of antigen retrieval (AR) was required for doublecortin (DCX) detection. In this case, tissues were heated to 93°C for 20 min in a hot water bath in 1x Reveal Decloaker (BioCare Medical RV100M) in H_2_O_2_ prior to endogenous peroxidase block. All tissue sections were incubated overnight at 4°C with corresponding primary antibody (refer to Table 1 for a complete list of antibodies): DCX (Santa Cruz Biotechnology, Cat#: sc-8066), Sox2 (Santa Cruz Biotechnology, Cat#: sc-17320) and MAG (Cell Signaling, Cat#: 9043P). To ensure specificity of the primary antibody, control tissues were incubated with antibody diluent instead of primary antibody in each series of staining. The chromagen exposure time was then based on the presence of positive control staining (mouse tissue) and a lack of signal appearing on tissues exposed to secondary antibody only. Optimal primary antibody dilutions were determined by conducting immunohistochemistry with serial dilutions of each primary antibody on positive control tissues (mouse brain). Images were obtained with a Zeiss camera. For each antibody, all tissues of both species were immunostained simultaneously to eliminate batch-to-batch variability in antibody concentrations, incubation times, or chromogen exposure. All sections were stained with hematoxylin (Hematoxylin Ready-to-use; Invitrogen) before mounting on slides. Hematoxylin only stained tissues went through a series of 5 min washes and stains in H_2_O_2_, hematoxylin, H_2_O_2_, then Tris pH 7.4. All images were acquired using identical settings to prevent light, exposure or other subtle microscopy differences. Counts for Sox2 positive cells were conducted on Zeiss images taken at middle and posterior hippocampus brain regions at 20x magnification. Images were imported into Photoshop CS6 where gridlines were used to aid in the manual counting of all Sox2 positive cells within the field of view. Two-three brain sections from each animal were counted, pooled and averaged.

### Dopamine ELISA

NMR brain homogenates were processed as described in *Tissue Homogenization* methods section; 15 μL brain lysate was used. The protocol was followed as per manufacturer's instructions (Rocky Mountain Diagnostics, Inc, Colorado Springs, Colorado, USA).

### Statistics

Each age cohort for all immunohistochemical and protein analyses contained 4–6 animals; both males and females were included. Data are expressed as mean ± standard error of the mean (SEM). To compare values across developmental age, one-way analysis of variance (ANOVA) with Bonferroni *post-hoc* analysis was used. For direct species comparison, significance was determined with Student's *t*-test. All data were analyzed using GraphPad Prism version 6.0c for Mac OS X, GraphPad Software, San Diego California, USA, www.graphpad.com/. Data were considered statistically different at *p* < 0.05.

## Results

### Naked mole-rats are born with large brains and exhibit an extended postnatal growth period

**Figure 1:**
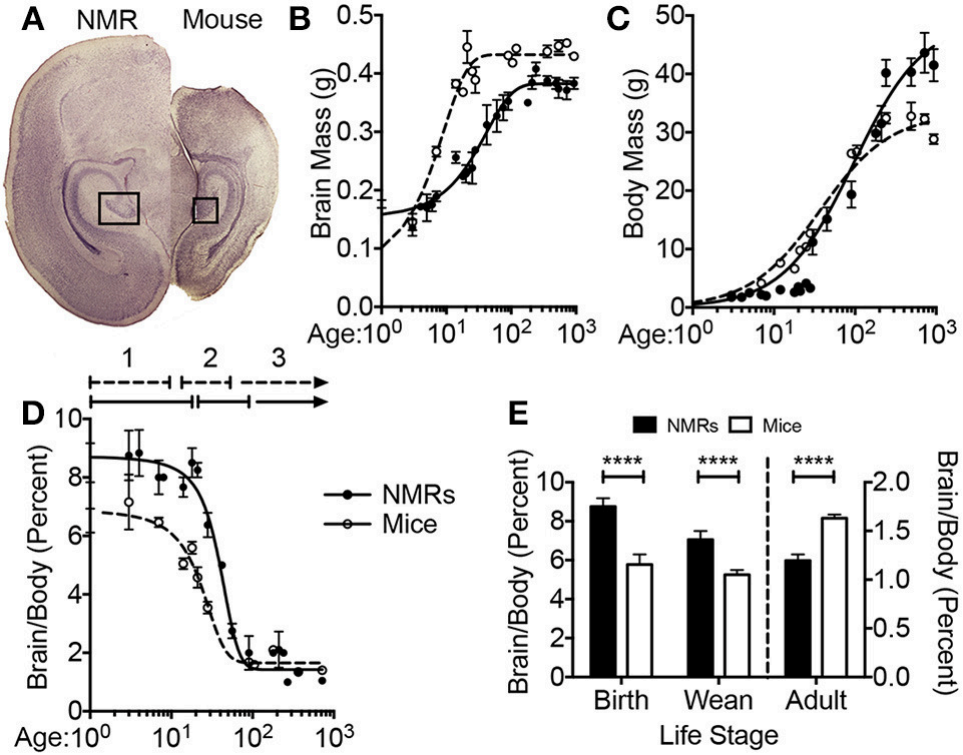
**Naked mole-rat brain growth significantly differs from mice**. **(A)** Newborn naked mole-rat (NMR) brains are twice as large as newborn mouse brains; shown are 4x images of 30 μm coronal brain sections stained with hematoxylin. The rectangle outlines the dentate gyrus of the hippocampi from each respective species. **(B)** Plotting brain mass against age reveals a dramatically different brain growth rate between species. Mouse brains are ~17% of adult mass at birth but by 2 weeks attain 90% of adult brain mass. NMRs brains are ~41% of adult size at birth but do not reach 90% adult mass until 3 months of age. **(C)** The age of somatic growth surge is similar between species and may reflect the age at which they begins to eat solids (3 weeks in mice vs. 3–6 weeks in NMRs). **(D)** Plotting the percentage of brain mass to body mass against age illustrates 3 distinct phases of development (1, 2, 3), that differ in length of time between species. The first static phase (1) last twice as long in NMRs as mice (4 and 2 weeks, respectively). The second phase (2) lasts 2 weeks in mice but 9 weeks in NMRs giving rise to the final phase of maintained body and brain mass beginning at 4 weeks in mice and 3 months in NMRs. **(E)** NMR brains are signifianctly larger than mice, even when normalized to body mass, through the age of weaning. However, adult NMR brains are significantly smaller than mice especially when accounting for body mass. (Student's *t*-test ^****^: *p* < 0.0001).

At birth, the average NMR pup weighs 34% more than the average mouse pup (1.793 ± 0.066 vs. 1.275 ± 0.007 g, respectively; *p* = 0.001; Figure 1, Table 2). Strikingly, NMR brain weight at birth is more than double that of the mouse brain (154.6 ± 3.3 mg and 73.3 ± 6.2 mg, respectively, Student's *t*-test *p* < 0.0001; Table 2, Figures 1A,B). Although NMRs are born larger (somatic and brain size) than mice, in the early postnatal days, mouse brain, and body growth is more rapid allowing mice to quickly surpass NMR brain and body mass measurements; in the first 2 weeks mice increase brain mass and body mass at rates 3 and 5-fold greater than NMRs, respectively (mouse brain: 22 mg/day; NMR brain: 7 mg/day; mouse body: 454 mg/day; NMR body: 88 mg/day). By 2 weeks of age mouse brains have reached 90% adult size, which contrasts greatly to that of NMRs that require 3 months (Figure 1B). The final adult brain and body mass differ significantly between species where NMRs, interestingly, have smaller brains (NMR: 376 ± 7.9 mg and mouse: 433 ± 2.7 mg; Table 2), but larger bodies than mice (NMR: 42.67 ± 2.22 and mice: 26.28 ± 0.73 g; Table 2) resulting in brains contributing a smaller proportion of body size (1.2 ± 0 0.1%, 1.6 ± 0.0% respectively; *p* < 0.0001; Table 2, Figure 1). Plotting NMR brain mass/body mass data uncovered that the three key growth phases of development common among many species (Kobayashi, 1963) differs between mice and NMRs (Figure 1D). The first phase is a static phase, and lasts twice as long in NMRs as mice (4 weeks and 2 weeks, respectively). The second phase is marked by a growth spurt resulting in an abrupt decrease in percent brain/body mass occurs. At the beginning of this phase mice are more than twice as large as NMRs (3.22 ± 0.22 g and 8.51 ± 0.53 g, respectively; Figure 1D). This growth spurt phase lasts until 9 weeks of age in NMRs, but is completed by 4 weeks in mice (Kobayashi, 1963; Figures 1B–D). During the final phase, species maintain body and brain mass. The experimental measures from our mice are in good agreement with those initially reported for the three distinct mouse growth phases (Kobayashi, 1963). Further, at each stage of development (birth, wean, and adulthood) the percent brain/body mass is significantly different between species (Figure 1E). At birth and wean, NMR brains are significantly larger than mice (even when normalized to body weight). In contrast, adult NMRs have significantly smaller brains (total brain mass and when normalized to body weight). Collectively these data indicate that while NMRs may be born with precociously developed brains as evidenced by larger mass, their postnatal growth is slower, they require more time to achieve adult size than short-lived mice, yet their brains do not grow as much as mice after they are born.

**Table 2:** **Interspecies comparison of life history traits between mice and NMRs**.

	**Mouse**	**Naked mole-rat**	**Significance**
Average gestation period (days)	21	71	N.D.
Age at weaning^*^ (weeks)	3	3–6	N.D.
Age at sexual maturity (weeks)	6–8	24^#^	N.D.
Maximum lifespan (years)	4	>31	N.D.
Body weight birth (g)	1.28 ± 0.01 (*n* = 6)	1.79 ± 6.6 (*n* = 27)	*p* = 0.0010
Brain weight birth (mg)	73.3 ± 6.2	154.6 ± 3.3	*p* < 0.0001
Percent brain/body mass birth (%)	5.6 ± 0.5	8.75 ± 0.4	*p* = 0.0020
Body weight at weaning^*^ (g)	8.51 ± 0.53 (*n* = 9)	3.22 ± 0.22 (*n* = 9)	*p* < 0.0001
Brain weight at weaning^*^ (mg)	393.9 ± 10.6	230.3 ± 5.8	*p* < 0.0001
Percent brain/body mass at weaning^*^	4.8 ± 0.3	7.4 ± 0.5	*p* < 0.0001
Body weight adult (g)	26.28 ± 0.73 (*n* = 37)	42.67 ± 2.22 (*n* = 35)	*p* < 0.0001
Brain weight adult (mg)	433.0 ± 2.7	376.3 ± 7.9	*p* < 0.0001
Percent brain/body mass adult	1.6 ± 0.0	1.2 ± 0.1	*p* < 0.0001

N.D: Not Determined;

* Begin to eat solids and decreased dependence on nursing;

# *Observation from our laboratory. Mean ± SEM*.

### NMR brains appear precociously developed at birth, but they maintain neurogenic potential longer than mice

**Figure 2:**
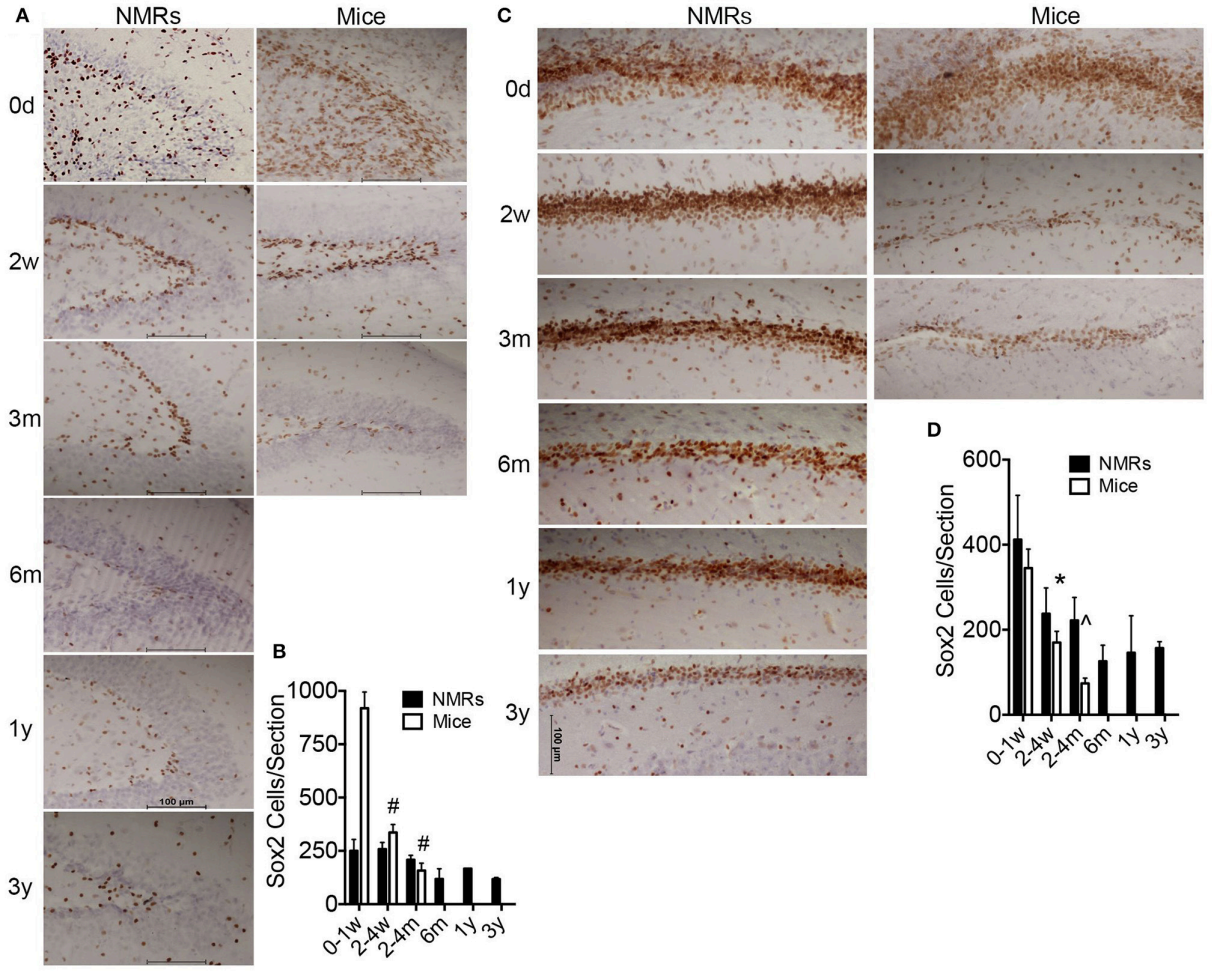
**Newborn naked mole-rat brains are born with fewer Sox2 positive neural stem cells than mice yet maintain neurogenic capacity longer than mice**. **(A)** Dentate gyrus from naked mole-rat (NMR) and mouse brains immunostained with Sox2 antibody and hematoxylin counterstain illustrate that newborn NMR dentate gyrus contains fewer Sox2 positive cells than newborn mice and instead appears more organized with clear lamination, even more-so than 1 week old mice. **(B)** Quantification of Sox2 positive cells indicate that at birth mice contain ~5x more Sox2 positive cells than NMRs. The level of Sox2 significantly decreases in mice and levels in the 2–4 weeks old cohort are similar between species. Levels of mouse Sox2 expression are significantly higher in newborns than all other cohorts (ANOVA, a: *p* < 0.0001). Significant differences in NMRs are not observed. **(C)** Images of subventricular zone from NMRs and mice stained with Sox2 reveals a similar density of Sox2 positive cells between species at birth; however mice quickly deplete this stem cell population as very few cells are evident by 2 weeks of age while NMRs maintain steady levels even into young adulthood. **(D)** Quantification of subventricular zone Sox2 positive cells indicate that mice quickly deplete this stem cell population as a significant decrease is observed between birth and the older cohorts in both regions (ANOVA, #*p* < 0.0001; ∧*p* = 0.0003; ^*^p = 0.0043). Significant differences among NMR ages are not observed.

**Figure 3:**
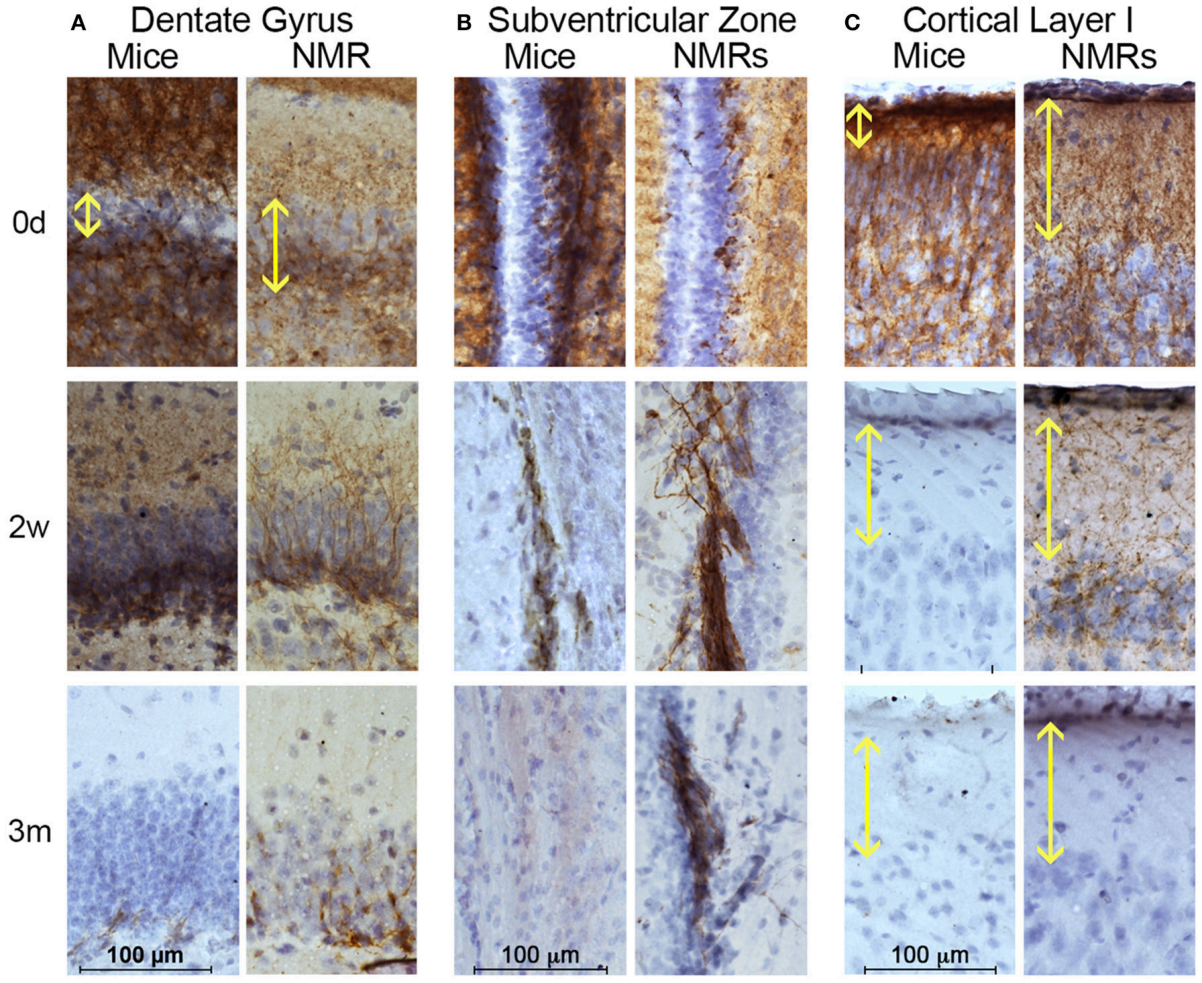
**Naked mole-rats are born with more mature brains than mice yet maintain neuronal plasticity longer than mice**. Naked mole-rat (NMR) and mouse brains were stained with newborn neuron marker doublecortin (DCX) and counterstained with hematoxylin. Immunostaining indicates that NMRs maintain DCX-positive cells in neurogenic **(A)** dentate gyrus and **(B)** subventricular as well as **(C)** cortex longer than mice. **(A)** Specifically, the newborn NMR dentate gyrus contains clearly defined cell layers with lower DCX-staining than newborn mice indicating greater *in utero* brain development in NMRs than mice. In both species, DCX-positive dentate gyrus cells become rare by 3 months of age. **(B)** Immunoreactivity is evident in NMR subventricular through 6 months of age, but becomes rare in 3-month-old mice. **(C)** Mice display poorly defined cortical organization until 2 weeks of age. In contrast, even at birth, NMRs exhibit a well-developed cortical layer I (yellow double arrow). By 2 weeks of age, mouse cortical DCX immunoreactivity is lost but it persists in NMRs. **(B)** Scale bar: 100 μm.

We utilized immunohistochemistry to determine morphometric differences between mouse and NMR brains, and examine neurogenesis and differentiation (Figures 2, 3). The transcription factor Sox2 (Sex determining region of Y chromosome (Sry)-related high mobility group box2) is necessary for maintaining neural progenitor identity (Uwanogho et al., 1995; Graham et al., 2003). It is one of the earliest expressed transcription factors in the developing central nervous system (CNS) and its expression is maintained in adult neural stem cells. We observed striking differences between species in each of the neurogenic zones in regard to expression of Sox2. NMRs are born with a well-defined dentate gyrus (DG) molecular layer with significantly fewer cells expressing Sox2 cells than observed in mice (Figures 1A, 2A,B). In stark contrast, nearly all DG cells in newborn mouse brains are Sox2 positive, and do not appear in the characteristic laminated organized compact cellular layers until 1–2 weeks of age (Figures 2A,B). The number of Sox2 labeled DG cells throughout development is relatively stable in NMRs; however, mice are born with significantly higher levels of proliferating cells that rapidly decrease in the first postnatal weeks (*p* < 0.0001; Figure 3B). Both species are born with comparable levels of Sox2 expressing cells in the ventricular zone (VZ). The distinct anatomy of the VZ breaks down during postnatal development (Tramontin et al., 2003); as such we measured levels of Sox2 in the subventricular zone (SVZ) throughout postnatal development. This level significantly drops in mice in the first postnatal weeks of life (*p* = 0.0043; Figures 2C,D); however, NMRs experience a gradual decline throughout development and their levels do not significantly change between birth and 3 years of age (*p* = 0.2676; Figures 2C,D).

The microtubule-associated protein doublecortin (DCX) is highly expressed in differentiating neurons and often is referred to as a “migratory marker” as it facilitates neuronal migration in these newborn cells (Francis et al., 1999; Horesh et al., 1999). At birth, NMR and mouse brains contain high levels of DCX-immunoreactivity in both proliferative zones (Figures 3A,B). In keeping with the Sox2 findings, mouse DCX expression is higher than NMRs at birth, but rapidly decreases in both the DG and SVZ whereas NMRs experience a gradual decline in expression (Figures 3A,B). In the DG, granular layer cells are evident already in newborn NMRs, with DCX-immunoreactive neurites extending projections through this cell layer. However, as seen with Sox2, mouse DG appear much less defined at birth and do not become laminated until 2 weeks of age (Figure 3A); these data parallel that of Sox2 (Figure 2).

The outermost cortical layer, cortical layer I, is the final cortical layer to develop in mammals (Angevine and Sidman, 1961) and contrasts greatly between the species studied here (Figure 3C). In mice, cortical layer I does not become clearly defined until 2 weeks of age, and loses DCX expression by 3 weeks. In contrast, the DCX cortical histology suggests the NMRs are born with a more organized cortical arrangement and more developed cortical development at birth. Nevertheless, the period of cortical maturation is longer than that of mice (Figure 3C) and extends 6 weeks postnatally as evidenced by persistent DCX expression in layer I at this age. Collectively the interspecific differences in three different brain regions and two antibodies support the premise that despite their precociously developed brains at birth, NMRs preserve stable neurogenic potential far longer than do mice indicative of a protracted developmental period.

### Cytoskeletal measures indicate that NMR neurons remain in a developmental phase for at least 6 months

**Figure 4:**
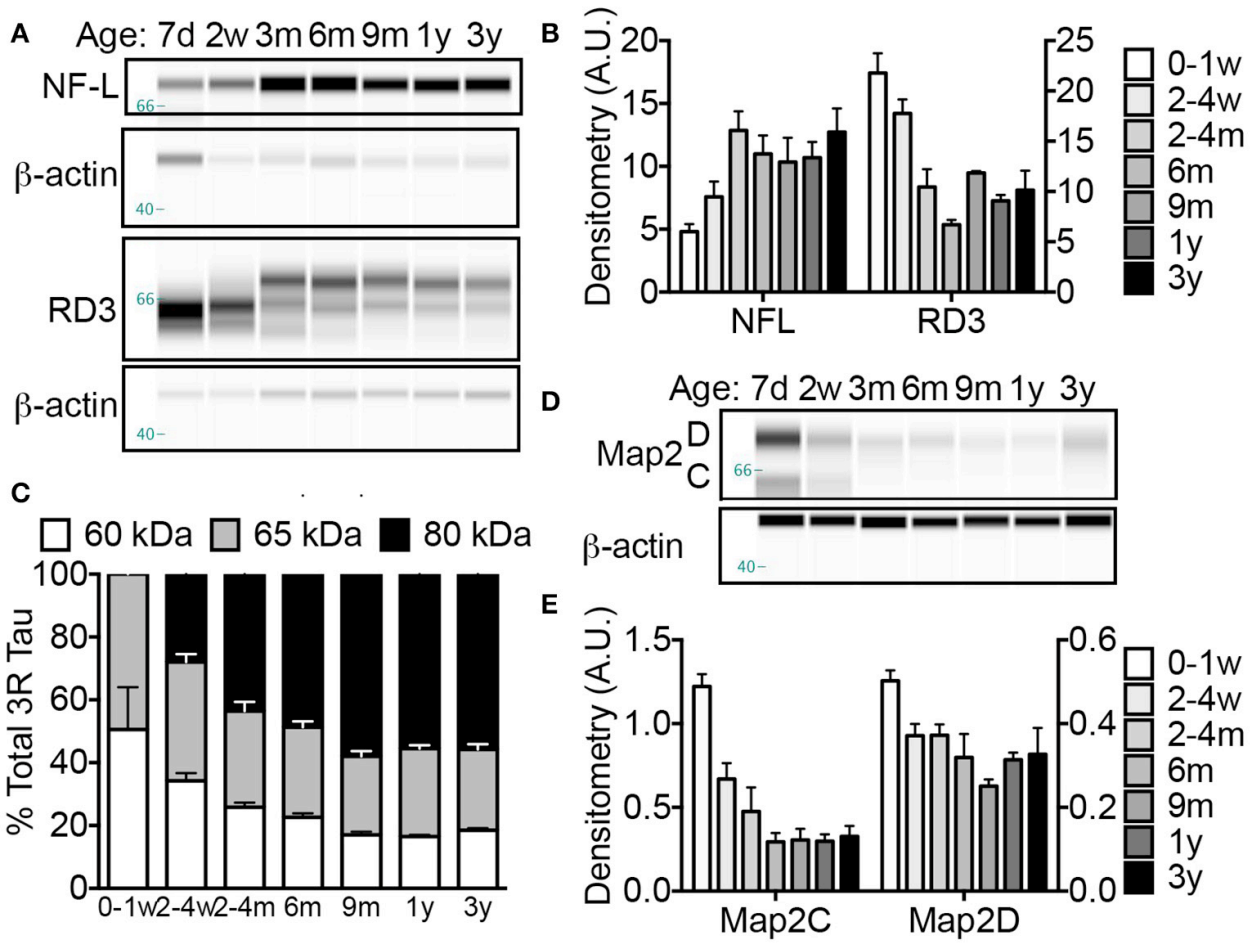
**Naked mole-rat brains attain stable adult levels of key neuronal markers at 6 months of age. (A)** Artificial capillary electrophoresis (CE) immunoblot reveals that while levels of axonal protein neurofilament light chain (NF-L), increases dramatically during the first 3 months of postnatal development, a simultaneous decrease in axonal three-repeat tau protein occurs. **(B)** Densitometric normalization to β-actin and statistical analyses indicates that both NF-L and RD3 levels change significantly during the first 3 years of life (ANOVA, *p* = 0.0002; *p* < 0.0001, respectively). Significance is reached with both axonal markers between birth and 2–4 months with an increase in NF-L (168%; *p* = 0.0002) and decrease in RD3 (52%; *p* < 0.0001). **(C)** Tau protein isoform distribution also changes with age as indicated by a transition in RD3 tau isoform expression from 1:1 low molecular weight 60:65 kDa during the first week of life to more prevalent high molecular weight tau 80 kDa species accounting for 50–60% of total RD3 protein at ages 6 months and older. **(D)** CE immunoblots were generated by probing naked mole-rat brain homogenates with an antibody directed against total microtubule associated protein 2 (Map2) dendritic proteins. **(E)** Analysis of densitometric normalization to β-actin loading control indicates a significant decline in Map2C and D during the first 3 years of life (ANOVA, *p* < 0.0001, and *p* = 0.0010, respectively). The expression of Map2C decreases immediately by 2–4 weeks (ANOVA, *p* = 0.0043) while the decrease in Map2D does not reach significance until 6 months (*p* = 0.0074). (d, days; w, weeks; m, months; y, years).

Neuronal cytoskeletal maturation has been well characterized in common laboratory rodents. In mice and rats, light, medium and heavy chain neurofilament (NF) proteins (NF-L, NF-M, and NF-H, respectively) all increase rapidly after birth and reach adult levels during the first postnatal weeks of life (Schlaepfer and Bruce, 1990; Kure and Brown, 1995). In contrast, human brain does not reach an adult pattern of neuronal cytoskeletal protein expression until 2 years of age (Arnold and Trojanowski, 1996). The microtubule associated protein tau (MAPT) gene encoding the axonal tau proteins also undergoes developmental regulation marked by a transition from producing tau protein isoforms with poor microtubule binding affinity to higher molecular weight tau isoforms better equipped to stabilize the cytoskeleton (Goedert et al., 1989a,b; Goedert and Jakes, 1990). While mice and rats exclusively express tau proteins containing three microtubule repeat (3R) domains during development (Kosik et al., 1989), both NMRs (Figure 4) and humans maintain three-repeat tau even in the adult brain (Goedert et al., 1989b). Capillary electrophoresis (CE) with antibodies specific to NF-L and 3R tau isoforms revealed significant changes in each of these tau markers during the first 3 years of NMR life (ANOVA, *p* = 0.0002; *p* < 0.0001, respectively). A 33% increase in NF-L occurs between birth and 2–4 weeks of age, and by 4 months NF-L is 168% greater than newborns (*p* = 0.0002) and twice that of 2–4-week-old NMRs (*p* = 0.025; Figures 4A,B). Simultaneously, 3R tau decreases 52% between newborns and 3–4 months of age (*p* < 0.0001; Figures 4A,B). This age, 4 months, seemingly marks a milestone in axonal maturity as evidenced by non-significant changes in NF-L or 3R tau protein expression from 4 months to 3 years of age (the oldest age cohort assessed in this study).

Notably, a difference in the proportion of tau isoforms also occurs during development. The low molecular weight (60 kDa) tau isoform decreases throughout the first year from 50% at birth to <20% in all animals aged 9 months and older (Figures 4A,B). Simultaneously, a high molecular weight, 80 kDa tau protein becomes the predominant tau protein comprising 49 and 58% of total 3R tau at 6 and 9 months, respectively (Figures 4A,C). This 80 kDa tau remains the prevalent isoform resulting with a 1:1.4:3 ratio of 60:65:80 kDa RD3 tau in 3-year-old animals (Figures 4A,C). The persistence of 3R tau protein expression even in adult brains is more similar to humans than other rodents. Mice and rats exclusively express 3R tau during development; only isolated neurogenic regions maintain 3R expression in adult mice (Kosik et al., 1989; Bullmann et al., 2007). In contrast human brains maintain 3R expression at an equal proportion with the adult isoforms (Goedert et al., 1989b; Kosik et al., 1989; Goedert and Jakes, 1990).

The dendritic cytoskeletal protein, microtubule associated protein 2 (Map2) also exhibits developmental changes (ANOVA, *p* < 0.0001). Like other rodents, NMRs are born with high levels of neonatal Map2C/D isoforms that displays a rapid decrease in expression during the first 2 postnatal weeks [45 and 26% decline, respectively by 2 weeks of age (*p* = 0.0043 and *p* = 0.0638; Figures 4D,E)]. The decline in Map2D did not reach significance until 6 months (36% decrease; *p* = 0.0074; Figures 4D,E) compared to a complete loss of expression by 3 weeks in rats (Riederer and Matus, 1985). Notably these developmental milestones correspond to ~1.5% of maximum lifespan for both species, highlighting the critical importance of accounting for biological age, not chronological age, when comparing protein expression levels among species especially during developmental stages.

### NMRs display exuberant synaptogenesis followed by pruning

**Figure 5:**
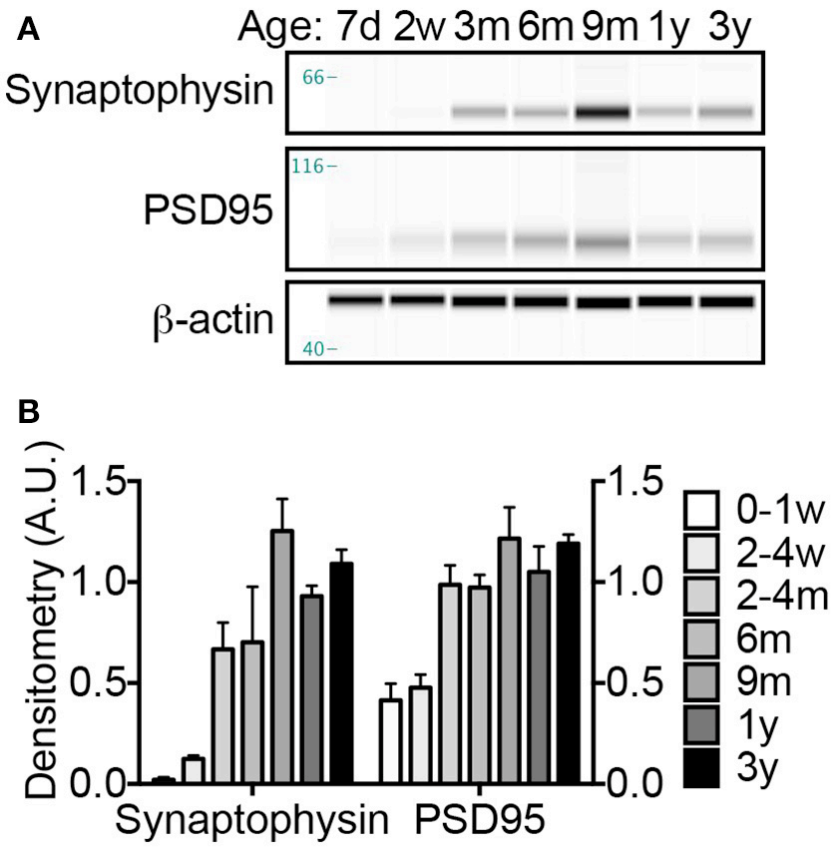
**Synaptogenesis increases dramatically at 2–4 months; refinement continues throughout the first 3 years of life. (A)** Synaptogenesis was assessed by immunoblotting brain homogenates with antibodies against presynaptic protein, synaptophysin, and postsynaptic protein, PSD95. **(B)** Densitometric values were calculated by normalizing to β-actin loading control. Significant changes in both synaptic proteins occur during the first 3 years of life (ANOVA, *p* < 0.0001 for both). A significant increase in both synaptophysin and PSD95 occurs between birth and 2–4 months (ANOVA, *p* = 0.0353, and *p* = 0.0002, respectively). ANOVA *posthoc* analyses indicate that animals over 2–4 months do not exhibit significantly different levels of either synaptophysin or PSD95; however Student's *t*-test between 2 and 4 months and older animals indicate a significant increase in synaptophsyin with the 9-month and 3-year-old NMRs (187% increase, *p* = 0.0326; 163% increase, *p* = 0.0089), but not on 1-year-old NMRs (*p* = 0.0936). Notably, levels of both synaptophysin and PSD95 decrease between 9 months and 1 year, 35 and 16%, respectively. (d, days; w, weeks; m, months; y, years).

During axonogenesis, as axons reach postsynaptic neurons the synaptic connections initiate pro-survival neurotrophic-signaling events to ensure the maintenance of these burgeoning neurons. The biochemical and morphological changes resulting from the communication between the incoming neuron (presynaptic neuron) and the dendrite (postsynaptic neuron) is referred to as synaptogenesis. Low levels of synaptogenesis begin *in utero*, but dramatically increase until 15 years of age in humans (Glantz et al., 2007). In contrast, these events occur rapidly in laboratory rodents, with a dramatic increase occurring between postnatal days 10–35 (Knaus et al., 1986; Sans et al., 2000). Using well-characterized markers of pre- and post-synaptic densities, synaptophysin and PSD95, respectively, we found NMR synaptogenesis to occurs much in the same fashion as humans with a burst of “exuberant synaptogenesis” that in subsequent ages becomes pruned. NMR synaptophysin levels did not significantly increase from birth until 2–4 months of age where a pronounced surge in expression occurred (305% increase; *p* = 0.0353; Figure 5). Levels again increased dramatically between the 2–4 month-old cohort and 9-month-old animals, though not significantly due to the high variance (88% increase; *p* = 0.07; Figures 5A,B). Levels did not increase after 9 months of age. As seen with synaptophysin, PSD95 levels increase dramatically between birth and 2–4 months of age (139% increase; *p* = 0.0002; Figures 5A,B). Neither synaptophysin nor PSD95 increase after 9 months of age, but interestingly were lower in 1- and 3-year-old NMRs (synaptophysin: 26 and 13%; PSD95 14 and 2%, respectively), suggesting synaptic refinement/pruning occurs after exuberant synaptogenesis.

### Tyrosine hydroxylase reaches adult level by 6 months of age, while dopamine increases until 2 years of age

**Figure 6:**
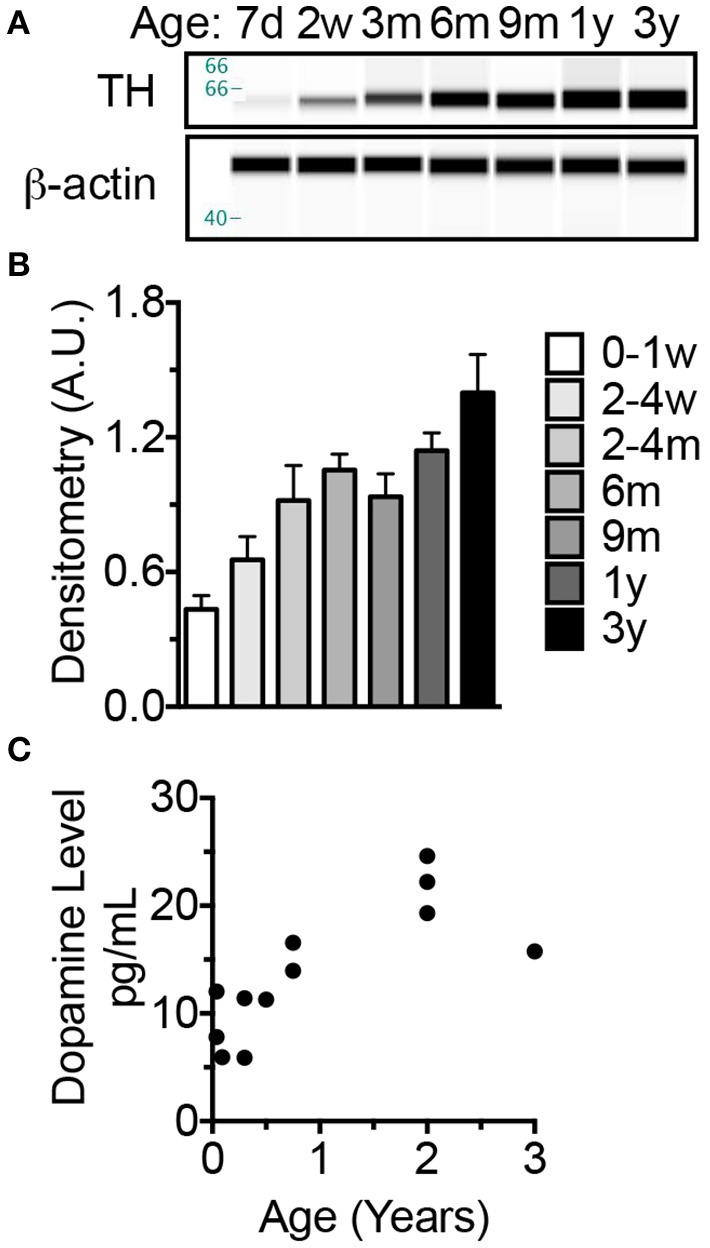
**The increase in dopamine trails that of tyrosine hydroxylase which, like long-lived primates exhibits an overshoot before decreasing to adult levels**. **(A)** Tyrosine hydroxylase (TH), a key enzyme involved in synthesizing monoamine neurotransmitters, levels were measured by capillary electrophoresis (CE) immunoblotting. **(B)** Statistical analyses on densitometric normalized values indicate a significance increase at 2–4 months of age (*p* = 0.0429). Levels do not significantly change between 4 months and 3 years. **(C)** ELISA measures of brain homogenate dopamine indicate an increase in levels through 2 years of age then a decrease at 3 years of age.

Concurrent with brain development, brain dopaminergic tone (experience-independent basal firing) rises in early development and exerts trophic roles including neuroblast division, cell migration, and synapse formation (Kalsbeek et al., 1988; Lankford et al., 1988; Gelbard et al., 1990; Todd, 1992; Tang et al., 2001). In as such, tyrosine hydroxylase (TH), the catalyst for the rate-limiting step in catecholamine biosynthesis, has been used as a marker to assess the density of dopaminergic afferents to the cerebral cortex. Developmental studies have shown a progressive increase in TH until 2 years of age in humans (Haycock et al., 2003) and non-human primates (Rosenberg and Lewis, 1995) before decreasing over the next several years. In rats, these events occur more rapidly and adult levels of TH occur at 4–5 weeks of age commensurate with the onset of breeding (Coyle and Campochiaro, 1976; Broaddus and Bennett, 1990b). Our assessment of TH levels in NMR brains reveals a progressive increase in expression between birth and 3 years of age (ANOVA, *p* < 0.0001) (Figure 6). Levels are 112% higher in 2–4-month-olds than newborns (*p* = 0.0429), and continue to increase through 3 years of age (223% increase from birth, *p* < 0.0001 (Figures 6A,B). In primates dopamine levels peak in the prefrontal cortex at puberty onset, 2–3 years of age (Goldman-Rakic and Brown, 1982; Plant, 1988) before declining to reach stable adult levels; humans also exhibit an “overshoot” during development reaching maximum levels at 9 years of age before decreasing (Haycock et al., 2003). In NMRs dopamine increases 4-fold from birth to 2 years before decreasing 28% at 3 years, indicative of an “overshoot” as seen in human and non-human primates (Figure 6C). Also similar to primates, the increase in dopamine trails that of TH (Figure 6C).

### NMR neuronal myelination is not completed until 1 year of age

**Figure 7:**
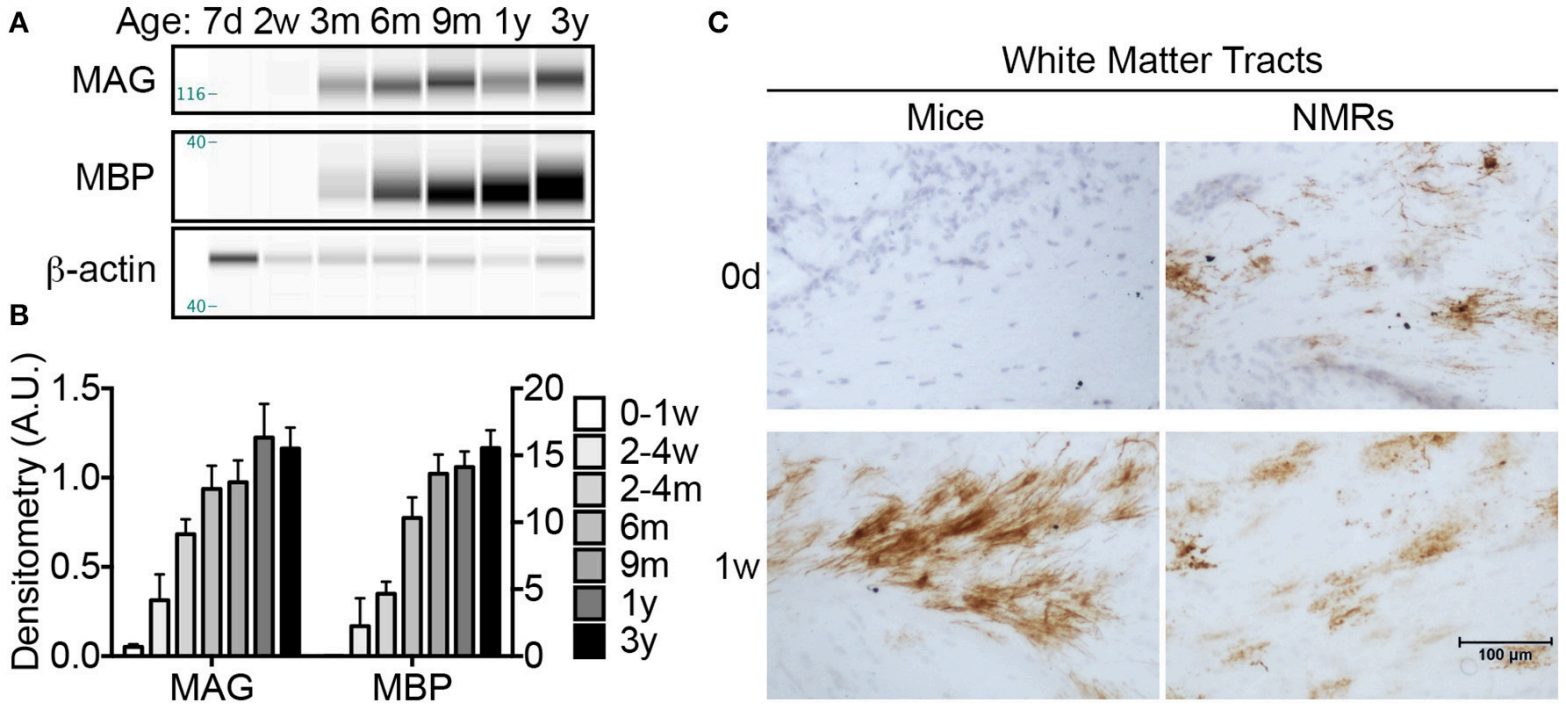
**Myelination begins ***in utero*** in naked mole-rat but not mice**. **(A)** Artificial immunoblots were generated with capillary electrophoresis by probing naked mole-rat (NMR) brain homogenates with antibodies directed against myelin associated glycoprotein (MAG) and myelin basic protein (MBP). Each isoform was normalized to β-actin. **(B)** Densitometric values were calculated by normalizing to β-actin loading control. Statistical analyses reveal significant changes during the first 3 years of life for both proteins (ANOVA, *p* < 0.0001 for both). MAG levels increase significantly from nearly undetectable in newborns to 1233% increase by 2–4 months of age (ANOVA, *p* = 0.0004). MAG increases significantly again between 2 and 4 months and 1 year of age (79%, *p* = 0.016). MBP expression was slightly delayed and a significant increase was not apparent until 6 months of age (*p* = 0.006), and another significant jump between 2 and 4 months and 9 months (192%, *p* = 0.0112). **(C)** Immunohistochemistry with anti-MAG antibody revealed that myelination initiates in white matter prior to birth in NMRs as evidenced by positive staining in newborn pups; however MAG immunostaining is not evident in mice until 1 week of age. (d, days; w, weeks; m, months; y, years).

To assess myelination, one of the final steps in neuronal maturation, we performed histological and biochemical assays with antibodies against MAG and myelin basic protein (MBP), two well-characterized proteins expressed by mature oligodendrocytes. CE on cerebral lysate indicate that NMR MAG levels are extremely low, nearly undetectable, until 2–4 months of age where a 1233% increase from birth is observed (*p* = 0.0004; Figures 7A,B). MAG expression continues to rise and significantly increases again between 2 and 4 months and 1 year (79% increase; *p* = 0.016). MBP expression is more delayed than MAG, and a significant increase in MBP does not become evident until 6 months (*p* = 0.006). By 6 months of age, levels are non-significantly (37%) lower than 3-year-olds, and 9-month-old and 1-year-old cohorts are within 14% of 3-year-old adults. Notably, neither MBP nor MAG show significant differences between 6 months and 3 years indicating that stable myelin expression has been achieved by 6 months of age (Figures 7A,B).

Since MAG expression was nearly undetectable with CE in newborn animals, we utilized immunohistochemistry to better determine the myelination profile of neonates. Histological assessment revealed that NMRs, but not mice, indeed express MAG at birth (Figure 7C). While expression is isolated to a limited number of cells in NMR white matter tracts, the presence of myelinated axons at in NMRs, but not in mice, is noteworthy and consistent with human brain development. MAG expression quickly increased in mice, and by 1 week of age high levels of MAG immunoreactivity was observed in white matter tracts of both species (Figure 7C).

## Discussion

**Figure 8:**
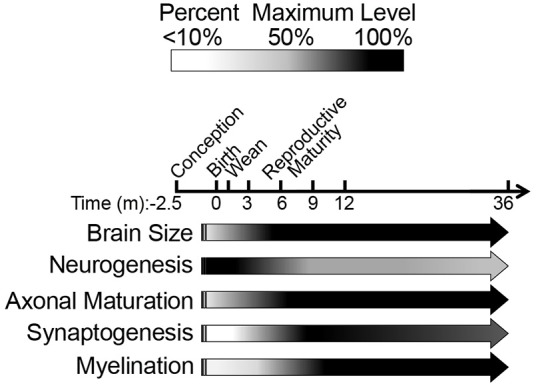
**Schematic summary of naked mole-rat brain development illustrates the major developmental milestones**. Our data, spanning from birth through the first 3 years of life, are represented by arrows. The black/white scale indicates percentage of maximum level (black) of each variable assessed, whereas the vertical lines before birth (gestation) represent hypothesized levels of each developmental measure based on newborn data. The majority of developmental changes occur during the first 3 months of age as indicated by a decline in NF-L and three-repeat tau expression. High molecular weight three-repeat tau predominates beyond 6 months of age. Protein markers of synaptogenesis become evident at 3 months of age and synaptogenesis become evident at 3 months of age, peak at 9 months, and decrease through 3 years of age. While NMR brains display myelination at birth, a steady increase continues through 1 year of age.

We questioned whether previous reports of neotenous traits in NMRs indicate a failure to mature, associated with delayed reproduction in subordinates (Jarvis, 1981; Pinto et al., 2010), or simply a difference in developmental heterochrony when compared to similar-sized rodents. Unlike commonly studied laboratory mice and rats, which complete brain maturation by 3 and 6 weeks of age, respectively, we found that NMR brain development tracks more closely with long-lived species (e.g., humans and non-human primates). Like human and non-human primate brains, NMR brains exhibit well-developed hippocampi and axonal track myelination at birth; postnatally NMR brains also undergo a prolonged maturation period with sustained neurogenesis into adulthood; and continue to express 3R tau protein isoforms even after brain growth is complete (Table 3). Significant changes in nearly all variables of axonal and dendritic maturation and myelination plateau by 6 months of age suggesting this age is key during NMR brain development (Figure 8 Summary). However, since levels of synaptic markers and dopamine continue to change until 3 years, we suggest 6 months marks a transition from adolescence into early stages of adulthood, and NMR brains may maintain low levels of plasticity even beyond this age.

**Table 3:** **Naked mole-rat brain ontogenesis more closely resembles humans than mice**.

**Human variable**	**Mice**	**NMRs**
Brain growth spurt predominantly *in utero;* 33% of adult size at birth Dobbing and Sands, 1979.	Brain growth spurt occurs postnatally (day 7); 16.9% adult size at birth Kobayashi, 1963; Watson et al., 2006.	Brain growth spurt predominantly *in utero*; 41.1% adult size at birth (Figure 1, Table 2).
Dentate gyrus development occurs predominantly *in utero* (~80%) Mathern et al., 1996, 2002.	Majority of dentate gyrus development occurs postnatally (80%) Schlessinger et al., 1975; Altman and Bayer, 1990; Diamond, 1990.	Dentate gyrus development predominantly *in utero*. Low postnatal DG neurogenesis (Figure 3).
Myelination begins *in utero* Brody et al., 1987.	Myelination begins postnatally (day 7) Jacobson, 1963.	Myelination begins *in utero*. Obvious myelination at birth (Figure 7).
Adult tau expression of 3R tau Goedert et al., 1989a; Kosik et al., 1989; Ksiezak-Reding et al., 1992; Köpke et al., 1993.	Low 3R tau expression in adults; restricted to neurogenic niche Kosik et al., 1989; Bullmann et al., 2007.	Adult 3R tau expression. Appreciable 3R expression in adult cerebrum. (Figure 5).

The over 3-fold longer gestation period observed in NMRs when compared to mice results in marked interspecies differences at birth in brain weight, myelination and mature cortical and hippocampal organization (Figures 2, 3, 7, Table 3). Collectively these data suggest that their long gestation period is devoted to both brain growth and neuronal maturation. Interestingly, myelination begins *in utero* in both humans (Brody et al., 1987) and NMRs, but does not commence until postnatal day 7 in rats (Jacobson, 1963) and mice (Table 2). We did not expect to observe axonal myelination at birth in NMRs since the pups do not independently explore their tunnel system in the first week of life. Living in highly cooperative communal groups, it is not uncommon for older NMR colony members, in addition to the dominant breeding female, to assist with pup rearing, and carry pups within the burrow system. As such, we believe the early myelination is consistent with their extended gestation period (like primates), and not a necessity for precocious movement immediately at birth. Similarly, a clearly laminated dentate gyrus is evident in newborn NMRs and humans (Mathern et al., 1996, 2002; Coe et al., 2003) and rhesus monkeys (Rakic and Nowakowski, 1981; Coe et al., 2003) but not in mice (Altman and Bayer, 1990; Diamond, 1990; Figures 2, 3). While examining NMR pups *in utero* would be difficult, and is beyond the scope of this study, NMRs, like humans, seemingly complete most neurogenesis and brain growth *in utero* as evidenced by large brains, developed hippocampi and myelinated axons at birth common to both species (Table 3).

While NMRs appear to allocate their extended *in utero* development to brain maturation rather than somatic growth, their larger brains at birth do not confer large adult brain size. In contrast, the African mole-rat rodent families (Bathyergidae and Heterocephalidae) are known to have the smallest brains of all mammals (Mace and Harvey, 1981). As such, NMRs do not fit the Workman model for predicting post conception neurodevelopmental milestones as adult brain mass and gestation length are critical to the model predictions (Workman et al., 2013). Though large cerebral capacity typically correlates with long life, and sociality drives evolution of large brains, NMR brain morphometry do not support these assertions. Rather the eusocial, preternaturally long-lived NMR, with a lifespan nearly eight times that of mice, has significantly smaller adult brains. Their evolution in a protected, dark, underground environment may explain the paradox. For example, many NMR characteristics resemble that of island dwelling species, a condition referred to as “Island Syndrome” (Lister, 1989; Adler and Levins, 1994). Small insular vertebrates experience low interspecific competition and often evolve morphological and behavioral changes due to the lack of predatory and reproductive pressures (Lister, 1989; Adler and Levins, 1994). NMRs share many of these traits including delayed sexual maturity, extended lifespan, high population density (up to 300 animals in a single colony), low aggressiveness toward conspecifics, decreased sexual dimorphism, and reduced basal metabolic rate (Jarvis, 1981; Goldman et al., 1999; Buffenstein, 2005). Further, the reduced size of adult brain mass in the African mole-rats is attributed to their reduced visual cortex, and considered an evolved response associated with the lack of visual cues in their dark underground milieu (Harvey et al., 1980). While their underground habitat may have offered them protection from predators and thereby allowed for longer developmental periods, both *in utero* and postnatally, the energetic constraints imposed by life under chronic hypoxia in this same environment may have contributed to smaller adult brain mass.

In seeming contradiction to their advanced brain maturation at birth is the NMRs maintenance of neural regenerative potential and extended cortical development. Other rodents significantly decrease neural stem cell proliferation within 2 weeks after birth (Rice and Barone, 2000; Babikian et al., 2010; Ben Abdallah et al., 2010), but NMRs contain statistically similar numbers of Sox2 cells in both neurogenic regions at all ages examined (Figure 3). A recent study reported that NMRs have lower levels of neurogenesis than adult mice (Penz et al., 2015). However, if adult neurogenic potential is compared to newborn levels within each respective species, the age-associated decline is far greater in mice than NMRs, with the latter species maintaining neonatal levels for at least 3-years of age. Indeed, the neural progenitor cell number we found is in keeping with observations of DG neurogenesis in adult wild-caught NMRs (Amrein et al., 2014). We propose that prolonged neurogenic capacity and delayed adult-like maturation co-evolved with the NMRs unique eusocial lifestyle as has been reported in the eusocial desert ant, *Cataglyphis albicans* (Seid and Wehner, 2009). These shared traits among eusocial invertebrates and the NMR would enable greater neuronal plasticity and may provide a neural basis for changes in social hierarchy and behavior with age.

Cell intrinsic (age, gene expression, etc.,) and extrinsic (trophic factors, extracellular matrix, etc.,) factors govern brain development, and dopamine exhibits both properties (Noisin and Thomas, 1988; Andersson et al., 2006). Dopaminergic markers (e.g., dopamine content and uptake, TH levels and activity) progressively increase during development; newborn rats are born with 10% of adult levels and monotonically increase to reach adult levels at 4–5 weeks (Coyle and Campochiaro, 1976; Broaddus and Bennett, 1990a) and 2–3 weeks of age in mice (Satoh and Suzuki, 1990; Ichinose et al., 2013). In humans and non-human primates developmental changes in the dopaminergic system closely track with the functional maturation of specific brain regions. For example in rhesus macaques, maximum levels of dopamine (Goldman-Rakic and Brown, 1982) and TH (Rosenberg and Lewis, 1995) are reached in the prefrontal cortex at 2–3 years of age, puberty onset (Plant, 1988), before declining to reach stable adult levels. In the entorhinal cortex, while dopamine does not greatly differ in layers I and VI, it peaks in layer III between 5 and 7 months of age (Erickson et al., 1998). In human striatum, dopaminergic markers increase during the first 9 years of postnatal life, peak, then decrease to stable adult levels (Haycock et al., 2003). In NMRs, TH expression reached that of adults by 6 months and dopamine expression progressively increases from birth to 2 years of age then decreases at 3 years. Also similar to humans, the increase in dopamine trails that of TH and does not peak until 2 years of age (Figure 6). Maturation of the dopaminergic system could correlate with lifespan and/or complex social structures as both are shared between humans and NMRs, but not mice or other rodents.

The microtubule associated protein tau is well recognized for its developmental regulation in multiple species from rodents to humans (Goedert et al., 1989b; Himmler, 1989; Kosik et al., 1989; Goedert and Jakes, 1990). For example, mice exclusively express three-repeat (3R) tau until postnatal day 8 where a rapid decline in its expression occurs (Kosik et al., 1989). Adult mice and rats do not express 3R tau except in elite subpopulations of dividing cells (Bullmann et al., 2007). During the first 6 months of life, NMR 3R tau expression significantly decreases; specifically lower molecular weight isoforms are replaced with higher molecular weight tau proteins. Nevertheless, NMRs maintain 3R tau expression through at least the first 3 years of age, which, mistakenly, could be considered “neotenous” if compared only between these rodents. However, adult human brains also express a 1:1 ratio of 3R and four-repeat (4R) tau (Goedert et al., 1989b; Kosik et al., 1989; Goedert and Jakes, 1990). Persistent expression of high molecular weight 3R tau indicates that NMR neuronal cytostructure tracks more closely with humans than mice or rats. This may be a feature of long-lived species or complex social behaviors. Since 3R tau binds microtubules with lower affinity than 4R tau, persistent expression even in adult NMRs may be important for neural plasticity which accompanies changes in social hierarchy and reproductive status associated with attaining dominance and becoming the breeding female (Holmes et al., 2011).

Concurrent with axonal maturation, neurons forming a long-lasting functional circuit must synaptically connect. Synapse number in laboratory rats increases from <1% in newborns to reach adult levels by 4 weeks of age (Sans et al., 2000). This process of synaptogenesis involves dendritic arborization and stabilization including the formation of pre- and postsynaptic densities. The significant increase in presynaptic synaptophsyin and postsynaptic PSD95 protein levels at 4 months of age in NMRs strongly suggests active synaptogenesis (Figure 5). Compared to the compressed 3-week timespan in other rodents (Paolicelli et al., 2011), these data again further emphasize the prolonged neuronal maturation in NMRs. Synaptic plasticity is crucial for cognitive function and maturation, and a failure in pruning contributes to disorders such as schizophrenia, fragile X, and autism in humans. During normal human cortical development, synaptophysin and PSD95 levels gradually increase after birth and peak at 6–10 years and then decrease over the next decade reaching “adult” levels by age 20 (Glantz et al., 2007). Rodent brains also require synaptic pruning as evidenced by behavioral deficits and syndromes similar to human fragile X and autism, when synaptic pruning is genetically modified (Comery et al., 1997; Irwin et al., 2000; Pfeiffer and Huber, 2009). However, synaptic pruning in rodents is difficult to assess due to their rapid brain maturation and compressed stages of development. Intriguingly, we found that both synaptophysin and PSD95 levels were higher at 9 months than any other age, including that of 1-year-old animals; this overshoot in synaptic marker expression may represent exuberant synaptogenesis, followed by a period of synaptic pruning. Notably, we observed the same overshoot in dopamine levels (Figure 6), and in numerous proteins involved in neurite outgrowth and neurotransmission recently published by our group. In our complementary study, cofilin-1, actin depolymerizing factor, spectrin alpha chain, septin-7, syntaxin-binding protein 1, synapsin-2 isoform IIB, and dynamin 1 all changed between the 2–3-year-old cohort and the 4–6 year-old cohort (Triplett et al., 2016). Differences in protein expression did not occur at any ages thereafter suggesting that final stages of synaptic refinement, brain plasticity, and maturation still likely are occurring even in these older animals. These observations warrant thorough investigation in future studies as NMRs may provide a novel animal model to study synaptic refinement and may be ideally suited to study behavioral consequences of improper synaptogenesis due to their unique eusocial colonial lifestyle.

Myelination occurs relatively late in nervous system development as it requires that neuronal axons have migrated and connected appropriately. These processes begin at postnatal day 7 in rats (Jacobson, 1963) and mice seen here (Figure 7); however myelination begins *in utero* in humans (Brody et al., 1987). NMRs are more similar to humans and non-human primates than laboratory rodents in this regard as we observed myelin protein expression at birth with an incremental increase slightly lagging that of axonal maturation (Figure 7, Table 3). Further, we saw that MAG expression slightly preceded MBP expression, which agrees well with their specific roles in myelination. MAG is a type I transmembrane glycoprotein expressed by myelin-forming cells that functions both in the formation and maintenance of myelin. MBP, however, begins to compact the cytoplasmic leaflets of the myelin bilayer only after myelin has been produced, and provides an explanation for the different ages of expression. Histologically, we found that NMR brains contain MAG expression at birth in select white matter tracks. A similar distribution in immunostaining did not occur in mice until 1 week of age, at which age mice rapidly increase MAG, and by 2 weeks of age, they appear similar to that of NMR brains indicating a rapid surge of myelination. Unlike the rapid spurt seen in mice, NMR myelination appears to progress gradually and continues to increase throughout the first 9 months of life, extending well beyond the 3 month timescale observed in mouse brain (Baumann and Pham-Dinh, 2001). These data provide additional evidence that NMRs are born with developmentally more developed brains, yet their postnatal brain maturation occurs slowly, not reaching adult levels until at least 6 months of age. While MRI-CT (magnetic resonance imaging-computed tomography) data suggest that NMR white matter tracts are within 50% of adult levels by postnatal day 8 (Seki et al., 2013), our biochemical and immunohistochemical data indicate that this does not occur until much later in development. Biochemically, MAG and MBP expression was <50% of adult in all cohorts younger than 2–4 and 6 months, respectively. Further, IHC and CE of MAG revealed very little immunostaining in 1-week-old animals followed by a striking increase in the 2-week-old cohort indicating the second week of life is a critical time for oligodendria expansion. We are not sure of the gender or size of the 8-day-old NMR from the previous study (Seki et al., 2013), so we cannot speculate as to why our data differ, but anticipate that sex, litter size, and number, in addition to the colony size and animal weight could possibly account for this discrepancy as initial litters display more rapid growth than later litters in established colonies (O'Riain and Jarvis, 1998). None of the animals in our study were from first litters to eliminate this variable.

### Conclusion

Our in depth investigation of NMR postnatal brain development reveals that this extraordinarily long-lived species gives birth to pups with more developed brains than mice in regard to mass, morphology and myelination. Despite more developed brains at birth than observed in mice, postnatally, NMRs exhibit protracted brain maturation more similar to that observed in long-lived species, than of short-lived rodents (e.g., mice and rats). Strikingly, NMRs do not fit mouse models for neurodevelopment (Workman et al., 2013), but instead track with human parameters if age-adjusted against maximum lifespan (i.e., humans have ~4-fold longer maximum lifespan and gestation period). If we consider that NMRs develop and age at a rate four-times faster than humans, many of our measures track closely with this estimate including levels of TH, dopamine, and synaptogenesis. While it may be tempting to consider that NMR brain development is not more protracted, but simply a shift relative to total post-conceptional age, our data do not support this premise. For example, a newborn NMR (71 days gestation period) does not resemble a 7-week-old mouse (21 days gestation and 50 days postnatal) in any measure. Our data, however, indicate that NMR brain development occurs more similarly to long-lived species. For example, NMRs and humans maintain expression of 3R tau isoforms even into adulthood, whereas mice only express 3R tau during development. Perhaps other markers of mouse neoteny cannot be applied uniformly among rodents but instead need to account for developmental period as well as maximum lifespan. Indeed, our studies on NMR brain have consistently found that when compared to mice, NMRs express high levels of many proteins typically considered “neotenous” including NMDA receptor subunit GluN2D (Peterson et al., 2012b), neuregulin (Edrey et al., 2012), and phosphorylated tau (Orr et al., 2015). While the use of traditional laboratory models has yielded a breadth of information regarding brain development, the field currently still lacks a model with critical parallels to human brain development (Watson et al., 2006). These include behavioral endpoints, peak brain growth spurt and myelination. We suggest that the NMR may be a useful model organism to better understand these events due to their long gestation, ~4–5 fold greater longevity than that predicted on the basis of body size and extended postnatal brain development.

## Author contributions

MO and RB conceived the study and planned experiments. RB provided the naked mole-rats used in this study. MO, VG, and AS performed experiments. MO and VG performed statistical analyses and analyzed data. MO, VG, AS, and RB discussed and interpreted the data acquired in this study. MO wrote the first draft of the manuscript and RB edited the manuscript. RB provided the funding needed for this study.

## Funding

We gratefully acknowledge funding for this study to RB from the Glenn and Owen's foundation. MO was supported by the National Institute of Aging training grant (T32AG021890).

### Conflict of interest statement

The authors declare that the research was conducted in the absence of any commercial or financial relationships that could be construed as a potential conflict of interest.

## Abbreviations

NMR: naked mole-rat
DG: dentate gyrus
SVZ: subventricular zone
VZ: ventricular zone
MAPT: microtubule associated protein tau
3R: three-repeat tau
4R: four-repeat tau
MAP: microtubule associated protein
MAG: myelin-associated glycoprotein
MBP: myelin basic protein
NF: neurofilament
NF-L: neurofilament light chain
TH: tyrosine hydroxylase
DCX: doublecortin
NMDA: N-methyl-D-aspartate
GluN2D: NMDA Receptor 2D
CE: capillary electrophoresis
g: grams
mg: milligrams
d: days
w: weeks
m: months
y: years
SEM: standard error of the mean
MRI-CT: magnetic resonance imaging-computed tomography.
